# Children Sleeping Together

**Published:** 1862-04

**Authors:** 


### CHILDREN SLEEPING TOGETHER.

As soon as children reach their seventh year, various good purposes would be subserved by their sleeping apart; indeed the neglect of this arrangement has cherished feelings, and has ultimately led to early vicious practices, alike destructive of the health of the body and the purity of the heart—to become, years before adolescence is passed, a source of physical and mental maladies sometimes, from which death itself is a welcomed deliverance.

Here, a branch of the subject opens a field of investigation at once wide and important, but one which requires so much judgment in the handling, that it is a debatable question, whether or not it should be left unexplored. Some have treated the subject, but with such want of discretion, that Carpenter, of England, one of the best physiologists of the age, hesitatingly records his opinion and regret at the evil tendencies of the publications made.

During the later "teens" of youth, certain debilitating occurrences take place in the early morning hours in the rather un-

[84]

sound or dreamy sleep which precedes the waking up, which, if allowed to continue unchecked, waste away the vigor and flesh and strength of the body, eventually impairing the mind itself, causing an unaccountable depression of spirits, a distressing nervousness which declines sometimes into a settled stupor or deplorable idiocy; others again become furious maniacs, according to the various constitutions and temperaments of the persons affected.

This malady does not occur to all young or unmarried persons by any means, but it does afflict many to a greater or less extent; not especially hurtful to any, if it does not occur oftener than two or three times a month; that much is perhaps a necessity; beyond that, it soon becomes a disease, with the manifestations already described.

These occurrences take place as a result of nature's exuberance, or as a consequence of practices, of the ill effects of which, those who engage in them, are most profoundly unconscious. These effects are sometimes traced to their legitimate causes by those who are unusually bright or thoughtful, the practices are at once abandoned, and the effects cease to be observed to any specially hurtful extent. But multitudes of the young never have the practices nor the deplorable results presented to their minds as cause and effect, and hence they continue the practices, and thus aggravate the effects, until the bodily and mental condition are alike pitiable and deplorable. Many parents who have grown up virtuously, witness with deep concern the pallid faces of once ruddy children, the trembling fingers, the averted eye, the thinned flesh, and the melancholy features; on inquiry, the feet are cold, the limbs weak, the body chilly, the appetite indifferent, the general system irregular, and pictures of a deep decline, wake up the affections in the deepest alarm; but nothing is

[85]

complained of; there is no pain, no suffering, while both parent and child may be alike unconscious of the existence of the causes of such effects; and while they are hoping for a change for the better to take place, for the renovating influences of the gladdening spring-time, or the bracing power of the coming fall, the malady may have run on to a condition irremediable, and the victim passes to the mad-house or to the grave.

The writer knew a gentleman of wealth, who had two sons; the elder was sent to a distant institution of learning at the age of eighteen years. He was a youth of manly bearing and of high promise. His attainments were unusual for one of his age; an estate was coming to him at his majority, which would yield him a revenue of twenty-three thousand dollars a year. His health began to decline. This was traced to practices into which he had been inveigled, of which no one could know any thing but himself. He was ignorant of their tendencies, and continued them until the morning debilitations became a drain so exhaustive to the vital powers, that he grew pale and thin and nervous. In a few months his bodily elasticity was gone. In place of the habitual courtesy, the high-bred deportment, and the joyous abandon which once characterized him in a remarkable degree, there was a listlesness of demeanor, a slovenliness of person and dress, with a settled shade of deep melancholy. A mental depression seized upon him, which it seemed impossible to remove by the amusements and diversions which commonly have great attractions for the young. In short, he became idiotic eventually, lost the power of speech, and now for nineteen years has not uttered a single word, nor is it at all like-

[86]

ly that he ever will, although thousands are spent every year in vain efforts for his restoration.

Some by the same means fall into consumptive disease and die in a few years; others become insane, and spend their weary lives in a lunatic asylum, or, in a moment of frenzied delirium, end their tortures and their existence by a suicide's guilty hand. Standard medical books abound in such deplorable narrations, but no public good could arise from varied repetitions. The causes and the consequences are the same, and the end uniformly deplorable. A very common cause is allowing children to sleep together, and when once the practices are commenced, sleeping together nourishes and cherishes them until they become an unquenchable and an unappeasable pleasure. There are, however, some so pure-minded, who have been so well brought up by worthy mothers, in being kept away from evil associations, that they have never learned the pernicious lessons, and learning them intuitively is a bare probability. One of the very best safeguards, in this direction, is never to allow your own children to sleep with the children of others, even for a portion of a single night. Your neighbor may be as pure and blameless as yourself, but never having had the attention directed to the point in question, may have been remiss in the matter of her children's associations, or may have had an overweening confidence in their correctness, by which the taint may have been introduced, and may have grown into a settled habit without any conception of its existence ever having entered the imagination.

While sleeping together generally founds the habits in question, on the part of children and youth, sleeping alone affords the amplest opportunity of unbridled indulgence. Hence, if parents observe a decided manifestation of symptoms which have been enumerated, very

[87]

especially if connected with a seeking for solitude, with a desire to be alone, and sleep alone, the best means for ascertaining certainly that such habits exist, and at the same time, without wounding the self-respect, is to occupy the same bed for several nights in succession, in wakefulness, and the manifestations will be made in a most unmistakable manner, either as to the habits or the exhaustions. It is barely possible that a week shall pass without such exhibition. In either case, take your child into your confidence, and without blame or accusation, without any charge of criminality, but in an incidental and affectionate manner, say: "My child, I noticed something last night not uncommon with youth, and as you may not know the nature of it, perhaps it might be best for me to tell you all about it, because sometimes persons become deranged by it, or kill themselves." Then make the communication in such a way that your child may not feel like a criminal, and at the same time, may be deeply impressed with the nature of the results which will follow a continuance of the practice. If the exhaustions occur without any connection with the practices, as is the case in multitudes of instances, from an exuberance of nature's fires, and hence without an iota of blame to the subject of them, or whether they have arisen from the practices in question, the remedy is the same, which is to adopt such means as will most effectually break up the occurrences, which can be done with comparative ease when the parent and the child work together, without there being any necessity of calling in a physician, which would be more or less embarrassing to all parties.

In using the methods about to be proposed, it is again particularly urged, as an important means of arriving more speedily at valuable practical results, that parents take special pains to show that they do not regard these things as degrading, as the result of criminal influences; for they are not so, necessarily, but simply as an exuberance of nature or of health, over

[88]

which the party affected has no control. It will have a good influence in every way, to let it be seen that it is regarded simply as an attack of illness, such as bilious fever, rheumatism, neuralgia, and the like. In this manner, the self-respect of the party is not wounded, and the way is opened for confidences and the interchange of affectionate sympathies, as in the case of other sicknesses, between parents and children, with the result that the means employed will be undertaken with a spirit and hopefulness which would not exist under other circumstances.

It may be proper here to advise parents of the existence of books of a vile character, which are scattered broadcast over the land, under various names, but the general term, 'Physiology," is used to designate them. Under the pretense of explaining the physiology of the parts implicated, the passions and the curiosity are stimulated by illustrations and descriptions, and plates which no person of proper self-respect would ever be seen examining, and which can not possibly fail of having a corrupting and a degrading influence. The reading of such books is fraught with unmixed evil. The first design is to effect a sale of the book, the next is to mislead the reader, to work upon the fears, under the guise of "frankness," and "humanity," and when the mind is wrought up to a pitch of frenzied terror of impossible things, to propose "a remedy efficient and certain in all cases, by reason of the fact that the writer has had a life-time's experience and success, his studies having led him to investigations and remedies, in the prosecution of which a fortunate accident led to a series of discoveries of incalculable value, and that out of sympathy for the misfortunes of the young, this mode of living usefully has presented itself."

Sometimes, a man constitutes himself a "Society," under some other taking designation, "Benevolent," "Humanitarian," and the like,

[89]

and advertisements, carefully worded, containing expressions of great charity and disinterestedness, are scattered every where, carrying with them influences and results of a most deplorable character. The young are lured by them, first to apply for a circular or a book, sent, sometimes, absolutely free of all cost, with the expectation, but too frequently realized, that the next thing will be an application for advice. The symptoms are inquired for, then comes a letter enlarging upon the "very dangerous character of the case, but that, possibly, by prompt and very close attention, the evil may be warded off, although it will necessarily be very expensive."

Let it suffice to say, that city physicians are constantly applied to, anonymously, for advice. The general tenor of the letter is, after expressing, in the most lively terms, the mortification and self-abasement felt, with the strongest self-accusatory confessions, to say that having expended ten, fifty, a hundred dollars, and sometimes several hundred, in taking medicines of some individual or society, they find that, if benefited at all, it was but transiently, and that their troubles have returned. But while in correspondence with their wily advisers, great care was taken by them to impress on the mind of their victim the disreputable nature of the ailment, to the following result, in cases not a few: Means have been devised by the victims to procure money in an unauthorized manner; how many "tills" of employers have been invaded; how many parental treasuries have been surreptitiously depleted; how many false representations of needs have been preferred to indulgent and confiding parents, may never be known; but that these things have been done on an extensive scale, and that it has been the first step towards actual crime in many a youth, only ending in the penitentiary or the gallows, is certainly true; and that the number is multitudinous, can not be reasonably questioned for a moment. It could not be otherwise; the youth is alarmed, and sensitive as the young are to such developments, it is not wonderful that their brains are literally racked with devices to procure the ways and means for meeting the remorseless demands of the "practitioners" named; and that those who have no means of making or earning money, should resort to subterfuges, and to untruthful representations, or to conduct actually criminal, in order to raise the requisite amount, requires no special stretch of the imagination to conjecture. Hence, the judicious use of the suggestions of these pages may save, and will save many a youth from a fate worse than that of death itself.

These pernicious books, with a great show of frankness, give various formulas for making the requisite medicines to be taken; but these are, for the most part, known to be inert. This is adroitly done, for the victim in all cases will certainly try what can be done, in the hope of avoiding the necessity of a confession or exposure, or the payment of a considerable fee. When, however, the means prescribed fail, the next impression is that it must be a very aggravated and threatening case, and at once the way is prepared for a consultation and the subsequent demand for a high fee.

Let it be distinctly remembered there is no absolute cure for the exhaustions in question short of marriage, old age, or death. There are some remedies which repress them while they are taken, and for a short time after, but the trouble returns, and with it the necessity for renewed applications for advice and remedies, and with them renewed extortions.

No medicines should ever be used by the persons themselves, on their own judgment and responsibility; nor should they be advised by any but the educated physician. Hence, there will be proposed in these pages only such remedial and moral means as can be employed with perfect safety and with an infallible good effect, more or less decided, according to the fidelity with which they are attended to, and according to the force of character and will of the person employing them.

Let it be remembered that these early morning exhaustions often arise in debility, without any other cause, as swollen feet and ankles arise from debility from various causes, and do not exist of themselves as an evidence of the last stages of consumption, although it is a symptom which generally attends the last stages of that disease, but it is not a "sign" of it, for it exists in other cases of debilitating disease when the lungs are not at all consumptive. The occurrences in question, when excessive, are of themselves simply a "sign" of debility, but greatly aggravate it, and thus they reäct on one another. Debility produces them, they increase the debility, and in process of time a single occurrence so depresses mind and body, that for hours and even days afterward, the mind is utterly incapable of concentrating itself properly on any business, while the body is absolutely unfit for any efficient employment, except at a sacrifice of its well-being.

But there is another enormity to which attention is specially invited. The remedies employed, which are repressive, are believed by many intelligent medical practitioners to be hurtful if injudiciously used; that is, used too largely, or too long; hurtful to the extent of a life-long disablement; and it is easy to conceive that a young or inexperienced, or reckless, or unprincipled practitioner may be tempted, by a variety of selfish considerations, or be led by actual incapacity, to employ these medicines to the hurtful extent named. Nor is this all. In the event of failure of success, men have been found vile enough to advise as the only means of cure, when marriage was impracticable, unlawful associations, which is but the opening of the flood-gates of vice, leading to the ruin of all that is noble and virtuous and pure; and how greedily the mind would lay hold of the excuse of the necessity of the thing, backed by the counsels of a medical adviser, all heedless of the utter selfishness of an act which brings a mere conjectural good to self, at the expense of an irreparable ruin to another! No one, it is repeated, can fail to see how willingly the mind would lay hold on such an apology as a satisfactory reason for greedily embracing the tempting alternative. It was in this connection that one of the most eminent medical writers has expressed his "regrets to be obliged to remark that some recent works which have issued from the medical press, contain much that is calculated to excite, rather than to repress the propensity, and that the advice sometimes given by practitioners to their patients is immoral as well as unscientific." And it may be said without exaggeration, that of all the rash and reckless and demented (for the time being) creatures in the universe, the greatest, the highest in the scale is the person who allows a single indulgence out of honorable and legalized wedlock; for one error in this direction is but the opening of flood-gates as resistless as an Alpine avalanche; it is the lighting up of desires as unsatisfiable and as remorseless as the Norwegian Maëlstrom. And not only so, a single error, committed in a much briefer time than is sufficient to express the sentiment, has been followed, in literally millions of cases, by the most revolting of human maladies, which, when even cured as far as external indications are concerned, still burrows in the system, poisoning the whole blood, and liable at any time to break out again like the smothered fires of a hatch-closed ship, to eat the flesh away, rottening even the bones, until life becomes a drawn-out torture. And in all cases when the system has been once impregnated with the virus, it never being eradicated, however perfect the health may seem to be, the effects are perpetuated to the offspring, to grow up with a worm at the root of life, a poison at the fountains of health, which sends out disease to every fiber of the system, cor-

(93)

rupting the blood and vitiating the whole body to such an extent, that it never knows an hour of pure health during its entire existence, although it may drag itself in weariness to the verge of three-score years and ten, literally years of sorrow and suffering! Such are some of the dangers to which the young are deliberately counseled to expose themselves, in the numberless cases where the medical adviser finds that all his vaunted remedies fail of even a temporary cure, and in recklessness and desperation, he counsels the passing of the Rubicon, with the results to the victim just described. And that under the circumstances, and with these views, which are true without exaggeration, it is the duty of every parent to know the position of his child in these regards, can not for one single moment be questioned by any rational mind.

For a further consideration of the subject, with the absolutely costless, perfectly safe and generally efficient treatment, see the Editor's book on "Sleep." $1 25.

